# Clinical and Genetic Spectrum of Patients with Pediatric-Onset Epilepsy: Insights from a Single-Center Study

**DOI:** 10.3390/genes16060624

**Published:** 2025-05-24

**Authors:** Hilmi Tozkir, Semih Asikovali, Esra Bozgeyik, Gurkan Gurbuz

**Affiliations:** 1 Department of Medical Genetics, Faculty of Medicine, Tekirdag Namik Kemal University, Tekirdag 59010, Türkiye; semihasikovali@nku.edu.tr; 2 Department of Medical Biology, Faculty of Medicine, Adiyaman University, Adiyaman 02040, Türkiye; ebozgeyik@adiyaman.edu.tr; 3 Department of Electroneurophysiology, Vocational School of Health Services, Istanbul Rumeli University, Istanbul 34570, Türkiye; gurkan.gurbuz@rumeli.edu.tr

**Keywords:** epilepsy, genetic testing, NGS, WES

## Abstract

**Objective:** Epilepsy, a common neurological disorder marked by recurrent seizures often starting in childhood, has a complex etiology. Advances in high-throughput sequencing now confirm that 70–80% of cases have a genetic basis. Accordingly, this study aims to evaluate the clinical relevance of genetic variations detected through epilepsy panels and whole exome sequencing (WES) in pediatric-onset epilepsy patients. **Methods:** For this study, we enrolled a cohort of pediatric patients involving 205 subjects with a preliminary diagnosis of epilepsy. Targeted next-generation sequencing panels for epilepsy and whole exome sequencing was performed using the NextSeq 500 platform. The results were analyzed with the QIAGEN Clinical Insight bioinformatic platform and were further confirmed and approved by the Human Genome Mutation Database and ClinVar databases. **Results:** In this study, an epilepsy panel was conducted in 138 patients, and whole exome sequencing was performed in 67 patients. No clinically relevant variants were identified in 29 (21.0%) patients who underwent the epilepsy panel and 27 (40.3%) patients who underwent WES. Variants were detected in 128 different genes in the epilepsy panel group and in 54 different genes in the WES group, with the frequency of these variants limited to one or two patients. **Significance:** In both the epilepsy panel and WES groups, variants in sodium channel proteins, specifically in the SCN1A, SCN8A, and SCN9A genes, were found to have a high frequency. Collectively, these findings suggest that sodium channel proteins may play an important role in epilepsy.

## 1. Introduction

Epilepsy is a neurological disorder characterized by the abnormal and excessive excitation of neurons in the brain that results in a persistent susceptibility to epileptic seizures and affects a significant number of individuals [1]. Epilepsy commonly occurs during childhood and presents as a clinically heterogeneous neurological disorder [2,3,4]. It is a complex disorder that encompasses multifactorial and diverse phenotypic characteristics. Despite the identification of certain fundamental disease pathways in epilepsy, its etiology remains elusive [5].

While it was once hypothesized that certain forms of epilepsy could have a genetic basis, the identification of specific mutations linked to epilepsy syndromes has now provided clear evidence supporting this genetic connection [6,7]. Current estimates suggest that 70-80% of epilepsy cases have a genetic etiology, while environmental factors like tumors and traumatic brain injury account for the remaining cases [3,8]. Early family and twin studies have demonstrated a considerable hereditary component in epilepsy. Particularly, monozygotic (MZ) twins have been identified to be far more concordant for epilepsy than dizygotic (DZ) twins [9]. Later twin analyses report similarly higher MZ:DZ ratios [10]. Epilepsy has a heritability of about 80%, with genetic generalized epilepsies showing slightly higher rates than focal types [11]. In addition to its heritability, epilepsy shares genetic risk with other neuropsychiatric conditions. Notably, epilepsy has been identified to have a positive genetic correlation with attention-deficit/hyperactivity disorder, particularly in focal epilepsy. However, it also has a negative correlation with intelligence, possibly due to intellectual disability [12].

The genetic basis of epilepsy may be either monogenic or polygenic. Genes or loci associated with primary epilepsy may also be linked to other neurological disorders in the genetics of epilepsy. Contemporary high-throughput sequencing technologies have enabled the identification of genes associated with epilepsy [3,7,13]. Particularly, next-generation sequencing (NGS) approaches, including whole exome sequencing (WES) and whole genome sequencing (WGS), have become increasingly common in recent years for identifying the genetic causes of certain diseases [14]. With the aid of the WES method, the analysis of single-nucleotide variants (SNVs) across all coding regions of the human genome has recently appeared as a cutting-edge, cost-effective method of choice for identifying potential disease-causing mutations. Particularly, WES has been effectively used to identify de novo mutations linked to neurodevelopmental disorders, including epilepsy [15].

Moreover, several genes have been implicated in the development of epilepsy syndromes. Epi25 Collaborative identified CACNA1G, EEF1A2, GABRG2, LGI1, and TRIM3 as most significantly associated genes with epilepsy syndromes [16]. In addition, in a cohort of Turkish patients, novel variations in ARX, ASPM, CACNA1A, CDK13, LGI1, MECP2, NF1, PCDH19, SCN1A, TSC2, and WDR45 genes have been reported [17]. Accordingly, in this study, we aimed to determine genetic variations in a cohort of epilepsy patients at a single center. For this study, epilepsy panels and WES analysis in patients diagnosed with pediatric-onset epilepsy were conducted.

## 2. Materials and Methods

### 2.1. Study Population, Sampling, and DNA Isolation

This study included 205 patients who were referred to the Department of Medical Genetics, Tekirdağ Namık Kemal University, with a preliminary diagnosis of epilepsy. The sample size of 205 patients was determined based on the availability of pediatric-onset epilepsy cases referred to our department over a 3-year period (2021–2023). This sample size was considered adequate to explore the distribution of rare variants detected by NGS techniques in a real-world setting. Given the exploratory nature of this study and the heterogeneity of epilepsy, we prioritized inclusivity to reflect the clinical spectrum encountered in routine practice. No prior power calculation was performed as the study was observational and descriptive, not hypothesis-driven.

Inclusion criteria: Patients aged 0–19 years referred to the Department of Medical Genetics at Tekirdağ Namık Kemal University with a preliminary diagnosis of epilepsy, confirmed by a pediatric neurologist through clinical findings and EEG/MRI. Only those who underwent an epilepsy panel or WES and had sufficient DNA quality and quantity were included.

Exclusion criteria: Patients were excluded if they had incomplete clinical data (such as missing EEG or MRI results), inadequate DNA quality, a known acquired cause of epilepsy (including post-traumatic epilepsy, tumors, or infections), a prior molecular diagnosis of a genetic syndrome unrelated to epilepsy, or if parental consent was not obtained.

Local ethics approval for this study was obtained from the Non-Interventional Clinical Research Ethics Committee of Tekirdağ Namık Kemal University, in accordance with the Declaration of Helsinki (Protocol No.: 2024.274.10.02). Blood samples of patients were collected using Vacutainer tubes with EDTA, and DNA was subsequently isolated from these samples. For DNA isolation, a QIAamp DNA Blood Mini QIAcube Kit (Qiagen, Hilden, Germany) was used. Isolations were carried out in accordance with the recommendations of the manufacturer. The purity and concentration of the isolated DNA samples were quantitatively assessed using a NanoDrop ND-2000 spectrophotometer (Thermo Scientific, Wilmington, USA). DNA samples that exhibited acceptable purity and concentration values were selected for subsequent analyses in this study.

### 2.2. Targeted Next-Generation Sequencing Panels for Epilepsy and Whole Exome Sequencing

Among the patients included in this study, some underwent genetic analysis via epilepsy panels, while others were analyzed using WES to obtain comprehensive genetic information. A total of 271 gene regions were analyzed in the epilepsy panel, and a list of genes was provided in Supplementary Table S1. Next-generation sequencing (NGS) libraries were first constructed using a QIAseq Human Exome Kit (Qiagen, Hilden, Germany). Following enrichment and quality control procedures, the samples were sequenced on the NextSeq 500 (Illumina Inc., San Diego, CA, USA) platform, achieving an average sequencing depth of 80x and 95.7% coverage, with paired-end reads of 150 bp. The resulting raw data were analyzed using the QIAGEN Clinical Insight (QCI) Interpret bioinformatics platform (Qiagen, Hilden, Germany). The in-silico prediction tools employed for the bioinformatics analyses included CADD, PROVEAN, SIFT, PolyPhen2, Human Splice Finder, and MutationTaster. Allele frequencies from population studies were obtained from the Exome Aggregation Consortium, the Genome Aggregation Database, and the 1000 Genomes Project. To evaluate the pathogenicity of variants, the Human Genome Mutation Database and ClinVar databases were utilized. The obtained data were analyzed according to the criteria established by the American College of Medical Genetics and Genomics (ACMG) [18]. All variants identified in the index cases were validated through Sanger sequencing.

### 2.3. Statistical Analysis

Descriptive statistical analyses were employed to determine the frequencies and distributions of the data. Percentages and proportions were utilized for the assessment of categorical data, while continuous data were evaluated using means and standard deviations. All calculations were performed with the aid of the GraphPad Prism 9 software.

## 3. Results

### 3.1. Study Population

Demographic and clinical characteristics of patients were presented in Table 1. In particular, a total of 205 patients were enrolled in the study, including 113 males (55.1%) and 92 females (44.9%). The mean age at the time of genetic testing was 7.46 ± 4.3 years, ranging 4 months to 19 years. Among these patients, 138 (67.3%) underwent epilepsy panel analysis, while 67 (32.7%) underwent whole exome sequencing. The clinical characteristics of the patients were assessed in terms of physical examination findings, MRI results, EEG findings, epilepsy syndromes, comorbidities, and the antiseizure medications used. Physical examination of the patients revealed that developmental delay was the most observed examination finding, occurring in 37.1% of cases. Additionally, microcephaly was identified in nine (4.4%) patients, while hypotonia was observed in five (2.4%) patients. According to the MRI findings, cerebral atrophy was observed in 7.8% of the patients. EEG findings indicated that 22.4% of the epilepsy patients had focal abnormalities, 19% exhibited generalized abnormalities, and 18% were diagnosed with epileptic encephalopathy. It was found that 13.7% of the patients had self-limited epilepsy syndrome, 12.2% had developmental and epileptic encephalopathy (DEE), and 5.9% had epileptic encephalopathy with spike-wave activation in sleep (EE-SWAS). Additionally, a significant majority of the patients (47.3%) had comorbidities. Among these comorbidities, developmental delay, intellectual disability (ID), and autism spectrum disorders were the most common. Lastly, the distribution of antiseizure medications used by the patients was evaluated, revealing that a significant majority (62.9%) were receiving at least one antiseizure drug. Among the antiseizure medications, the most commonly used were as follows: levetiracetam (41.5%), valproic acid (29.8%), clobazam (8.3%), carbamazepine (5.4%), and topiramate (2.9%).

### 3.2. Genetic Findings

Among the patients included in the study, no variant was detected in 29 (21.0%) patients subjected to an epilepsy panel and 27 (40.3%) patients subjected to WES analysis. The list of variants identified as clinically relevant was presented in Table 2 and Table 3. The frequency of previously identified variants in the patients of our study group was generally very low, typically limited to one or two patients. 

In the patients who underwent panel analysis, variants were identified in 128 different genes, with their chromosomal locations and amino acid changes detailed in Table 2. In the panel group, the most frequently identified variant was in the DNA polymerase subunit γ (POLG) gene, observed in seven patients (5.1%). This was followed by variants in the sodium voltage-gated channel α subunit 1 (SCN1A) gene in seven patients, sodium voltage-gated channel α subunit 9 (SCN9A) in seven patients, kinesin family member 1A (KIF1A) in five patients and contactin associated protein 2 (CNTNAP2) in five patients. According to the ACGM classification, variants were classified as follows: 6 benign, 7 likely benign, 27 likely pathogenic, 28 pathogenic, and 164 variants of uncertain significance. In the group that underwent WES, variants were identified in 54 different genes. A list of the variants associated with the patients is provided in Table 3. According to the ACGM classification, variants were classified as follows: 23 likely pathogenic, 7 pathogenic and 32 variants of uncertain significance. In the patients who underwent WES, the identified mutations were generally observed in only one patient; however, a variant in the WDR62 gene was found in two different patients. Of the genes in which variants were detected through WES in the patient cohort, 11 genes were also represented in the targeted gene panel analysis. Among these variations, only the c.407A>G (p.Gln136Arg) variant in the BTD gene was identified in both the panel analysis and WES. Variations were also identified in the SCN1A, SCN8A, and SCN9A genes within the WES group. This finding suggests that sodium channel proteins may play a significant role in epilepsy patients. 

## 4. Discussion

In complex diseases like epilepsy, which are heterogeneous and have an etiology that is not yet fully understood, efforts to elucidate its genetic basis using advanced technologies are becoming increasingly significant each day. The identification of clinically significant variants or mutations is at the forefront of disease management and therapeutic strategies. Therefore, findings obtained from large-scale sequencing methods such as NGS and WES not only introduce new genetic variations but also enhance our understanding of the pathophysiology in patients. A review of previous studies on epilepsy reveals a growing body of data from WES and NGS findings, particularly in recent years. These studies, aimed at detecting clinically significant variants, hold promise for the future in terms of both personalized medicine and early diagnosis. Targeted gene panels and WES are considered important for diagnosis and prognosis in patients with early-onset epilepsy [16,19]. Costain and colleagues reported a genetic diagnostic yield of 19% for epilepsy-targeted gene panels and 37% for WES in early-onset epilepsy patients [20]. In a meta-analysis conducted by Stefanski and colleagues, the overall diagnostic yield for epilepsy was analyzed to be 24% [21]. In another meta-analysis, the genetic diagnostic yield for epilepsy was found to be 23% for targeted gene panels and 32% for WES [22]. In our study, clinically significant variants were detected in the vast majority of patients who underwent an epilepsy panel and WES analysis. However, since the number of patients in our study group varied, a precise genetic diagnostic yield could not be calculated. Overall, it is noteworthy that in the WES group, there may be candidate genes or variants potentially associated with the epilepsy clinic. On the other hand, variations were detected in the sodium channel protein SCN1A, SCN8A, and SCN9A genes in our study group. Voltage-gated sodium channels are essential components that trigger the action potential in excitable cells like neurons. These channels are transmembrane proteins composed of one α-subunit, forming the central pore, and two smaller auxiliary β-subunits, regulating channel functions. Genetic alterations in the SCN1A gene, coding for the α-subunit of Na_V_ 1.1, are linked to various seizure-related disorders in humans [23]. The most well-known epilepsy phenotype associated with SCN1A is Dravet syndrome (OMIM: 607208), but it also causes several other epilepsy syndromes which are associated with various significant comorbidities. In addition, mutations in the SCN1A gene are also known to cause generalized epilepsy with febrile seizures plus (GEFS+) [24,25,26]. Similarly, SCN8A encodes the α-subunit of the Nav1.6 voltage-gated sodium channel involved in the initiation and propagation of action potentials within the nervous system [27]. More than 150 mutations in the SCN8A gene have been identified to be potentially associated with epilepsy. It has been reported that a large proportion of these mutations are de novo missense mutations [28]. Additionally, the R850Q mutation in the SCN8A gene has been shown to have a gain-of-function effect, suggesting that it may contribute to the development of early infantile epileptic encephalopathy [29]. The loss-of-function and gain-of-function mutations in voltage-gated sodium channel genes, which lead to various neuropsychiatric phenotypes, highlight the potential importance of these genes in diagnosis and prognosis. In the group analyzed with the epilepsy panel, the most variations were detected in the POLG gene (in seven patients, including five males and two females). The maintenance of mitochondrial genome integrity is essential for cellular energy production and metabolic homeostasis. The high-fidelity DNA polymerase γ is responsible for the accurate replication of mitochondrial DNA. The protein encoded by the POLG gene serves as the catalytic subunit of mitochondrial DNA polymerase γ. Mutations in POLG have been implicated in a range of neurological and myopathic disorders, as well as in phenotypes associated with premature aging [30]. Particularly, POLG mutations have been associated with Alpers–Huttenlocher syndrome (OMIM: 203700), the myocerebrohepatopathy spectrum, and juvenile and adult-onset myoclonic epilepsy (OMIM: 254770) [31,32]. Studies have reported that POLG mutations are frequently encountered in epilepsy patients, with 128 pathogenic POLG variants being associated with seizures [33,34,35]. It has been shown that in 84% of epilepsy patients, at least one of the pathogenic variants in the POLG gene, including p.Ala467Thr, p.Trp748Ser, and p.Gly848Ser, is present [33]. However, these pathogenic variants were not detected in our study cohort. This absence may reflect the considerable genetic heterogeneity of epilepsy, supporting our findings that suggest a more polygenic architecture, as multiple rare and potentially contributory variants were identified across different genes. It is crucial to note, however, that the presence of a variant in a gene does not necessarily imply that the gene is causally associated with the disease. There is often a significant gap between identifying a genetic variant and demonstrating its functional impact. Without experimental validation or segregation data, the pathogenicity of many variants remains uncertain. Furthermore, epilepsy associated with POLG is typically severe, and the majority of cases are resistant to treatment [36]. In our study group, one patient with a POLG variation was known to be using both valproic acid and levetiracetam, while another patient was using levetiracetam alone. A more in-depth evaluation of this situation is needed, along with detailed associative studies, to better predict the prognosis in patients who develop drug resistance. Collectively, although our findings also suggest that epilepsy is a polygenic disease, as variations were identified several different genes, the lack of parental data limits our ability to determine whether these variants act in concert or reflect de novo.

This study, conducted at a single center, has some limitations. One of the main limitations is the small sample size in the WES group. In addition, the uncertainty regarding the effects of the variations in both the epilepsy panel and WES groups on protein functions creates a gap in understanding the molecular mechanisms of epilepsy. Although the ACMG classification criteria [18] were used in our study, the presence of data such as LP, P, and VUS for some variants may lead to slight adjustments in categorization. Moreover, a major limitation of this study is the lack of parental DNA samples, which impairs the ability to determine whether detected variants are inherited or de novo. This distinction is crucial for accurate variant classification under ACMG guidelines. Without parental data, many rare variants cannot be confidently classified as benign or pathogenic, which leads to a higher proportion of variants being designated as of VUS. Studies show that the rate of VUS is higher in proband-only studies compared to trio analyses that include parental genomes. Moreover, the absence of parental genotypes limits the potential for novel gene discovery, as identifying recurrent de novo variants across patients is a key strategy for implicating new disease-associated genes in pediatric epilepsy. Moreover, although limited by a small sample size, this study offers valuable insights into the clinical use of genetic testing in epilepsy and highlights the need for larger studies to better define diagnostic yield and guide clinical decision-making. Lastly, our study is limited by the absence of structural bioinformatic analysis and the lack of use of tools such as AlphaFold, which could have clarified whether these mutations cluster within known functional domains or protein–protein interaction regions.

## 5. Conclusions

In conclusion, we have reported the clinical and genetic features of 205 pediatric epilepsy patients seen at a single center. Using epilepsy panels and WES, we identified rare variants in several genes, including POLG, SCN1A, SCN9A, KIF1A, and CNTNAP2, which were associated with specific clinical characteristics. Notably, variants in the SCN1A, SCN8A, and SCN9A genes, which encode sodium channel proteins, were found with high frequency in both groups, suggesting that disturbances in sodium channel function may play a significant role in the pathophysiology of epilepsy. Although a portion of patients showed no clinically relevant variants, the findings emphasize the importance of genetic testing in understanding the underlying causes of epilepsy, facilitating better diagnosis, and potentially informing future therapeutic strategies. Further research is needed to explore the clinical implications of these genetic variations and their role in personalized treatment approaches for epilepsy patients.

## Figures and Tables

**Table 1:** The demographic and clinical characteristics of the epilepsy patients included in this study.

Demographic and Clinical Characteristics		Epilepsy Panel (*n* = 138, %)	WES (*n* = 67, %)	Total (*n* = 205, %)
Gender	Male	74 (53.6%)	39 (58.2%)	113 (55.1%)
	Female	64 (46.4%)	28 (41.8%)	92 (44.9%)
Age	Mean ± SD	7.2 ± 4.15	8.6 ± 4.7	7.46 ± 4.3
Physical Examination	Developmental Delay	27 (19.6%)	49 (73.1%)	76 (37.1%)
	Microcephaly	2 (1.4%)	7 (10.4%)	9 (4.4%)
	Hypotonia	3 (2.2%)	2 (3.0%)	5 (2.4%)
MRI Findings	Cerebral Atrophy	4 (2.9%)	12 (17.9%)	16 (7.8%)
	Cerebellar Atrophy	1 (0.7%)	2 (3.0%)	3 (1.5%)
	Corpus Callosum Abnormalities	0 (0.0%)	1 (1.5%)	1 (0.5%)
	Cortical Dysplasia	1 (0.7%)	0 (0.0%)	1 (0.5%)
	PVL	2 (1.4%)	3 (4.5%)	5 (2.4%)
EEG Findings	Epileptic Encephalopathy	17 (12.3%)	20 (29.9%)	37 (18%)
	Focal Disorder	29 (21.0%)	17 (25.4%)	46 (22.4%)
	Generalized Disorder	31 (22.5%)	8 (11.9%)	39 (19%)
	Background Activity Abnormalities	1 (0.7%)	5 (7.5%)	6 (2.9%)
Epilepsy Syndrome	DEE	16 (11.6%)	9 (13.4%)	25 (12.2%)
	Etiology Specific Syndromes	1 (0.7%)	0 (0.0%)	1 (0.5%)
	Self-Limited Epilepsies	24 (17.4%)	4 (6.0%)	28 (13.7%)
	LGS	3 (2.2%)	5 (7.5%)	8 (3.9%)
	SWAS (ESES)	5 (3.6%)	7 (10.4%)	12 (5.9%)
	PME	1 (0.7%)	0 (0.0%)	1 (0.5%)
	LKS	1 (0.7%)	2 (3.0%)	3 (1.5%)
	JME	7 (5.1%)	0 (0.0%)	7 (3.4%)
	Other	14 (10.1%)	5 (7.5%)	19 (9.3%)
Comorbidity	Yes	39 (28.3%)	58 (86.6%)	97 (47.3%)
	No	99 (71.7%)	9 (13.4%)	108 (52.7%)
Number of Antiseizure Medications	1	95 (68.8%)	34 (50.7%)	129 (62.9%)
	2	17 (12.3%)	12 (17.9%)	29 (14.1%)
	3	3 (2.2%)	5 (7.5%)	8 (3.9%)
Antiseizure Medication	Valproic Acid	42 (30.4%)	19 (28.4%)	61 (29.8%)
	Levetiracetam	63 (45.7%)	22 (32.8%)	85 (41.5%)
	Carbamazepine	5 (3.6%)	6 (9.0%)	11 (5.4%)
	Clobazam	6 (4.3%)	11 (16.4%)	17 (8.3%)
	Topiramate	3 (2.2%)	3 (4.5%)	6 (2.9%)
	Other	22 (15.9%)	8 (11.9%)	30 (14.6%)

**Table 2:** The genetic variations in pediatric-onset epilepsy patients as determined by the epilepsy panel.

Panel Genes	Nucleotide Change	Amino Acid Change	Zygosity	ACMG Classification		Number of Patients (%)
ABAT	c.1214T>A	p.Leu405Gln	Heterozygote	VUS	PP3, PM2	1 (0.7%)
ABCA2	c.7175G>A	p.Arg2422Gln (p.Arg2392Gln)	Heterozygote	VUS	PM2, PP2	1 (0.7%)
	c.4238G>A (c.4238A>T)	p.Arg1413His (p.(Gln1413Leu))	Heterozygote	VUS	PM2, PP3, PP2	1 (0.7%)
ADAM22	c.1787+6G>A		Heterozygote	VUS	PM2, BP4	1 (0.7%)
AFG3L2	c.1683G>T	p.Gln561His	Heterozygote	VUS	PM2, PP2	1 (0.7%)
	c.1097A>G	p.Asn366Ser	Heterozygote	VUS	PM2, PP2	1 (0.7%)
AIMP1	AIMP1	p.Asn73Ser	Heterozygote	VUS	PM2, BP4	1 (0.7%)
ALDH7A1	c.622G>A	p.Ala208Thr	Heterozygote	VUS	PM2, PP3, PM1, PP2	1 (0.7%)
	c.1566-1G>T		Heterozygote	P	PM3, PVS1, PM2, PP5	1 (0.7%)
	c.1429G>T	p.Asp477Tyr	Heterozygote	VUS	PP3, PM2, PP2	1 (0.7%)
ALG13	c.1769T>G	p.Phe590Cys	Hemizygote	VUS	PM2	1 (0.7%)
AMACR	c.511C>T	p.Arg171Cys	Heterozygote	VUS	PM2	1 (0.7%)
AMT	c.1144C>T	p.Arg382Trp	Heterozygote	VUS	PM2, PP2	1 (0.7%)
	c.214A>G	p.Thr72Ala	Heterozygote	LB	PP3, PM1, PP2, BS2, BS1	1 (0.7%)
ANKRD11	c.5162C>T	p.Thr72Ala (p.Thr1721Met)	Heterozygote	VUS	PM2	1 (0.7%)
AP3B2	c.1234C>T	p.Arg412Trp	Heterozygote	VUS	PM2, BP1	1 (0.7%)
AP4B1	c.1160_1161delCA	p.Thr387Argfs*30	Heterozygote	P	PVS1, PM3, PM2, PP5	1 (0.7%)
	c.946A>G	p.Lys316Glu	Heterozygote	VUS	PM2	1 (0.7%)
	c.359A>G	p.Tyr120Cys	Heterozygote	VUS	PM2, PP3	1 (0.7%)
AP4E1	c.3152A>G	p.Asp1051Gly	Heterozygote	VUS	PM2	1 (0.7%)
AP4M1	c.1376G>C (c.1355G>C)	p.Arg452Pro	Heterozygote	VUS	PM2, PP3	1 (0.7%)
	c.973C>T (c.952C>T)	p.Arg325* (p.Arg318*)	Heterozygote	P	PVS1, PM3, PM2, PP5	2 (1.4%)
	c.772G>A (c.751G>A)	p.Asp258Asn (p.Asp251Asn)	Heterozygote	VUS	PM2	1 (0.7%)
AP4S1	c.229G>A	p.Glu77Lys	Heterozygote	VUS	PM2, PP3	1 (0.7%)
ARHGEF9	c.1142T>G	p.Met381Arg (p.Val381Gly)	Hemizygote	VUS	PM2	1 (0.7%)
ARID1B	c.4264C>T (c.4384C>T)	p.His1422Tyr (p.His1462Tyr)	Heterozygote	VUS	PM2, PP3	1 (0.7%)
ARSA	c.232C>T	p.Leu78Phe	Heterozygote	LP	PM2, PM1, PP3, PP2	1 (0.7%)
ASAH1	c.424C>A (c.376C>A)	p.Pro142Thr (p.Pro126Thr)	Heterozygote	VUS	PM2, PP2	1 (0.7%)
	c.595A>G (c.547A>G)	p.Lys199Glu (p.Lys183Glu)	Heterozygote	VUS	PM2, PP3, PM1, PP2	1 (0.7%)
ASPA	c.427A>G	p.Ile143Val	Heterozygote	LP	PM2, PM5, PM1, PP2, PP5	1 (0.7%)
ASPA	c.914C>A	p.Ala305Glu	Heterozygote	P	PM3, PM2, PP3, PM1, PP2, PS3, PP5	1 (0.7%)
ATP13A2	c.839A>G	p.Lys280Arg	Heterozygote	VUS	PM2	1 (0.7%)
	c.2404G>A	p.Gly802Ser	Heterozygote	VUS	PM2, BP6	1 (0.7%)
	c.3472C>T	p.Arg1158Cys	Heterozygote	LB	PM2, BP6, BS2	1 (0.7%)
BRAT1	c.1421C>T	p.Thr474Met	Heterozygote	LB	BP4, BP6	1 (0.7%)
	c.127G>C	p.Glu43Gln	Heterozygote	VUS	PM2, PP3	1 (0.7%)
BTD	c.407A>G	p.Gln136Arg	Homozygote	VUS	PM2, PM1, PP2	1 (0.7%)
	c.38_44delinsTCC	p.Cys13Phefs*36	Heterozygote	P	PVS1, PM3, PM2, PP5	1 (0.7%)
CACNA1A	c.6679C>T	p.Arg2227Cys	Heterozygote	VUS	PM2, PP3, PM5, PP2, BP6	1 (0.7%)
	c.6479G>A (c.6461G>A)	p.Arg2160Gln (p.Arg2154Gln)	Heterozygote	VUS	PM2, PP2, BP6	1 (0.7%)
	c.2010G>A (c.2007G>A)	p.Trp670* (p.Trp669*)	Heterozygote	LP	PVS1, PM2	1 (0.7%)
	c.4400+2T>C (c.4388+2T>C)		Heterozygote	LP	PVS1, PM2	1 (0.7%)
CACNA1E	c.5795G>A	p.Arg1932Gln	Heterozygote	VUS	PM2, PP2, BP6	1 (0.7%)
	c.6572_6574del	p.Glu2191del	Heterozygote	VUS	PM2, PM4, BP6	1 (0.7%)
	c.6920C>T	p.Thr2307Met	Heterozygote	VUS	PM2, PP2, BP6	1 (0.7%)
	c.1283G>A	p.Ser428Asn	Heterozygote	VUS	PM2, PP2	1 (0.7%)
CACNA1H	c.1084G>A	p.Asp362Asn	Heterozygote	VUS	PP3, PM2, BP6	1 (0.7%)
	c.3578T>A	p.Leu1193Gln	Heterozygote	VUS	PM2	2 (1.4%)
	c.3799C>T	p.Arg1267Trp	Heterozygote	VUS	PM2, BP6	1 (0.7%)
	c.3788C>A	p.Pro1263His	Heterozygote	VUS	PM2, PP3	1 (0.7%)
CASR	c.1199A>T	p.Asn400Ile	Heterozygote	VUS	PM2, PP2	1 (0.7%)
	c.3008C>T (c.2978C>T)	p.Thr1003Met (p.Thr993Met)	Heterozygote	VUS	PM2, PP2	1 (0.7%)
CC2D1A	c.1919A>G	p.Gln640Arg	Heterozygote	VUS	PM2, BP4	1 (0.7%)
CERS1	c.787G>A	p.Val263Ile	Heterozygote	VUS	PM2, BS2	1 (0.7%)
	c.925G>T	p.Val309Leu	Heterozygote	VUS	PM2, BP4	1 (0.7%)
	c.919G>A	p.Ala307Thr	Heterozygote	VUS	PM2, BP4	1 (0.7%)
CHD2	c.547A>G	p.Arg183Gly	Heterozygote	VUS	PM2, PP2	1 (0.7%)
CHRNA4	c.166G>A	p.Ala56Thr	Heterozygote	VUS	PM2, BP4	1 (0.7%)
CLCN2	c.2377G>A	p.Ala793Thr	Heterozygote	VUS	PM2	1 (0.7%)
CLN3	c.461-3C>A		Heterozygote	VUS	PM2, PP3	1 (0.7%)
	c.1133T>C	p.Ile378Thr	Heterozygote	VUS	PP3, PM2, PP2	1 (0.7%)
CLN6	c.255C>G	p.Phe85Leu	Heterozygote	VUS	PM2, PM1, PP2	1 (0.7%)
CLN8	c.46C>A	p.Leu16Met	Heterozygote	VUS	PM2, PM1	1 (0.7%)
CNTNAP2	c.3520G>A	p.Gly1174Arg	Heterozygote	VUS	PP3, PM2	1 (0.7%)
	c.2884G>A	p.Gly962Arg	Heterozygote	VUS	PP3, PM2	1 (0.7%)
	c.1562T>G	p.Met521Arg	Homozygote	LP	PP3, PM2	1 (0.7%)
	c.1030G>A	p.Gly344Ser	Heterozygote	VUS	PP3, PM2	1 (0.7%)
	c.181G>A	p.Gly61Ser	Heterozygote	VUS	PM2, PP3	1 (0.7%)
COL4A1	c.2399G>A	p.Gly800Glu	Heterozygote	VUS	PP3, PM2, PP2	1 (0.7%)
COX15	c.478G>A (c.-59-1G>A)	p.Gly160Ser (*)	Heterozygote	VUS	PM2	1 (0.7%)
	c.1000G>T	p.Val334Phe	Heterozygote	VUS	PM2	1 (0.7%)
CPT2	c.359A>G	p.Tyr120Cys	Heterozygote	P	PM3, PP3, PM2, PS3, PP1, PP5	1 (0.7%)
	c.157C>T	p.Pro53Ser	Heterozygote	VUS	PP3, PM2	1 (0.7%)
D2HGDH	c.1337C>T	p.Ala446Val	Heterozygote	VUS	PM2	1 (0.7%)
	c.1058A>G	p.Lys353Arg	Heterozygote	VUS	PM2, PP3	1 (0.7%)
DARS2	c.492+2T>C		Heterozygote	P	PM3, PVS1, PM2, PP5	1 (0.7%)
DHFR	c.144_145delGA	p.Asn49Serfs*6	Heterozygote	VUS	PM2	1 (0.7%)
	c.440C>T	p.Thr147Met	Heterozygote	VUS	PM2, PP3	2 (1.4%)
DNM1L	c.344C>T	p.Thr115Met	Heterozygote	LP	PP3, PM2, PM5, PP2	2 (1.4%)
	c.1442T>C (c.1403T>C)	p.Ile481Thr (p.Val468Ala)	Heterozygote	VUS	PP3, PM2, PP2	1 (0.7%)
DOCK7	c.1801-1593_1801-1590delTTAG (c.1832_1835del)	p.Val611Aspfs*32	Heterozygote	LP	PM3, PM2, PP5	1 (0.7%)
DPYD	c.187A>G	p.Lys63Glu	Heterozygote	LP	PM3, PM2, PP3, PS3, PP5	1 (0.7%)
	c.1905+1G>A		Heterozygote	LP	PVS1, PM2, PP5	1 (0.7%)
EARS2	c.1234C>T	p.Arg412Cys	Heterozygote	LP	PM3, PM2, PM5, BP4, PP5	2 (1.4%)
	c.497G>A	p.Arg166Gln	Heterozygote	VUS	PM2	1 (0.7%)
EFHC1	c.1307G>A	p.Arg436His	Heterozygote	LP	PP3, PM2, PM	1 (0.7%)
EIF2B1	c.808C>T	p.His270Tyr	Heterozygote	VUS	PM2, PP3	1 (0.7%)
EIF2B3	c.71C>T	p.Pro24Leu	Heterozygote	VUS	PP3, PM2	2 (1.4%)
EIF2B4	c.766C>G	p.Gln256Glu (p.Leu256Val)	Heterozygote	VUS	PM2, PP3	2 (1.4%)
FAM126A	c.968G>A	p.Arg323Lys	Heterozygote	VUS	PM2, PP3	1 (0.7%)
FARS2	c.361A>G	p.Thr121Ala	Heterozygote	VUS	PM2, BS2	3 (2.2%)
FOXG1	c.1372C>A	p.Pro458Thr	Heterozygote	VUS	PM2, PP2	1 (0.7%)
FOXRED1	c.449T>G	p.Leu150Arg	Heterozygote	VUS	PM2, PP3	2 (1.4%)
	c.580C>T	p.Arg194Trp	Heterozygote	VUS	PM2	1 (0.7%)
GABRG2	c.549-3T>G		Heterozygote	VUS	PM2, PP5, PP3	1 (0.7%)
GABRG3	c.586G>A	p.Gly196Arg	Heterozygote			1 (0.7%)
GALC	c.334A>G	p.Thr112Ala	Heterozygote	VUS	PM1, PP5, PP3, PP2, BS1, BS2	1 (0.7%)
	c.1042A>T	p.Thr348Ser	Heterozygote	VUS	PM2, PP3, PP2	1 (0.7%)
	c.1208A>G	p.Asn403Ser	Heterozygote	VUS	PM2, PM1, PP2	1 (0.7%)
GAMT	c.22C>A	p.Pro8Thr	Heterozygote	LB	PM2, BS2, BS3	1 (0.7%)
GCH1	c.274C>A	p.Leu92Ile	Heterozygote	LP	PM2, PM1, PP2, PP5	1 (0.7%)
GFAP	c.548G>A	p.Arg183His	Heterozygote	VUS	PP3, PM2, PP2	1 (0.7%)
	c.667G>C	p.Glu223Gln	Heterozygote	VUS	PM2, PP3, PP2, BP6	1 (0.7%)
GFM1	c.2005A>G	p.Met669Val	Heterozygote	VUS	PM2	2 (1.4%)
GLB1	c.176G>A	p.Arg107His (p.Arg59His)	Heterozygote	P	PM3, PP3, PM2, PM5, PM1, PP2, PS3, PP1, PP5	1 (0.7%)
	c.613G>A (c.469G>A)	p.Ala205Thr (p.Ala157Thr)	Heterozygote	LP	PM2, PM1, PP3, PP2	1 (0.7%)
GLDC	c.1210C>A	p.Pro96Ser	Heterozygote			1 (0.7%)
	c.1210C>A	p.Leu404Met	Heterozygote	VUS	PP3, PM2, PP2	1 (0.7%)
GNE	c.1721G>A (c.1628G>A)	p.Gly574Asp (p.Gly543Asp)	Heterozygote	VUS	PP3, PM2, PP2	1 (0.7%)
	c.2179G>A (c.2086G>A)	p.Val727Met (p.Val696Met)	Heterozygote	LP	PM2, PP3, PM1, PP2, PP5	1 (0.7%)
GRIA3	c.106A>G	p.Ile36Val	Heterozygote	VUS	PM2, PP2, BS2	1 (0.7%)
GRIN1	c.343C>T	p.Arg115Cys	Heterozygote	VUS	PM2, PP2, BP6	1 (0.7%)
	c.1406G>A (c.1343G>A)	p.Arg469His (c.1343G>A)	Heterozygote	VUS	PM2, PP2, BP6	1 (0.7%)
GRIN2A	c.1778-2A>G		Heterozygote	LP	PVS1, PM2	1 (0.7%)
GRIN2B	c.1249G>T	p.Val417Phe	Heterozygote	VUS	PM2, PP3, PP2	1 (0.7%)
HESX1	c.18G>C	p.Gln6His	Heterozygote	VUS	PM2	1 (0.7%)
HNRNPU	c.529C>T	p.Arg177Cys	Heterozygote	VUS	PM2, PP2, BP4	1 (0.7%)
HTRA1	c.245C>G	p.Pro82Arg	Heterozygote	B	PP2, BP4, BS1, BS2	1 (0.7%)
	c.844A>C	p.Ile282Leu	Heterozygote	VUS	PM2, PM1, PP2	1 (0.7%)
	c.451C>A	p.Gln151Lys	Heterozygote	VUS	PM2, PP2, BS2	1 (0.7%)
	c.961G>A	p.Ala321Thr	Heterozygote	LP	PP3, PM2, PM1, PP2	1 (0.7%)
IQSEC2	c.4325del	p.Pro1442Hisfs*53	Hemizygote	LP	PVS1, PM2, PP5	1 (0.7%)
KCNMA1	c.1335-6G>A		Heterozygote	VUS	PM2, BP4	1 (0.7%)
KCNQ2	c.997T>C	p.Phe333Leu	Heterozygote	LP	PM2, PM1, PP3, PP2	1 (0.7%)
	c.1118G>A	p.Ser373Asn	Heterozygote	VUS	PM2, PP2	1 (0.7%)
KCNQ3	c.917C>T	p.Ala306Val	Heterozygote	P	PS2, PP3, PM2, PM1, PP5	1 (0.7%)
KCNQ5	c.7C>A	p.Arg3Ser	Heterozygote	VUS	PM2, PP3, PP2, BP6	1 (0.7%)
	c.2834T>C (c.2777T>C)	p.Leu945Ser (p.Leu926Ser)	Heterozygote	VUS	PM2, PP2	1 (0.7%)
KCNT1	c.2096C>T	p.Thr699Met	Heterozygote	VUS	PM2, BP4	1 (0.7%)
KIF1A	c.3670C>T	p.Arg1224Trp	Heterozygote	VUS	PM2, PP2	1 (0.7%)
	c.2351G>A	p.Arg784Gln	Heterozygote	VUS	PM2, PP2	1 (0.7%)
	c.3887G>A	p.Arg1296His	Heterozygote	VUS	PM2, PP2, BP6	1 (0.7%)
	c.4852G>T	p.Gly1618Cys	Heterozygote	VUS	PM2, PP2, BP4	1 (0.7%)
	c.2704G>A	p.Glu902Lys	Heterozygote	VUS	PM2, PP2, BP4	1 (0.7%)
LAMA2	c.3037+2T>C		Heterozygote	LP	PVS1, PM2	1 (0.7%)
	c.2527C>T	p.Arg843Cys	Heterozygote	VUS	PM2	1 (0.7%)
LGI1	c.1516G>A	p.Ala506Thr	Heterozygote	VUS	PM2	1 (0.7%)
LRPPRC	c.2120C>A	p.Ser707Tyr	Heterozygote	VUS	PM2	1 (0.7%)
	c.3430C>G	p.Arg1144Gly	Heterozygote	VUS	PM2	1 (0.7%)
MBD5	c.1364C>A	p.Ala455Glu	Heterozygote	VUS	PM2	1 (0.7%)
MECP2	c.86A>G	p.Asp29Gly	Hemizygote	VUS	PM2	1 (0.7%)
MED17	c.1384G>A	p.Ala462Thr	Heterozygote	VUS	PM2	1 (0.7%)
MTFMT	c.797G>A	p.Arg266His	Heterozygote	VUS	PM2	1 (0.7%)
MTHFR	c.470G>A	p.Arg157Gln	Heterozygote	P	PM3, PP3, PM2, PS3, PP1, PP5	1 (0.7%)
NDST1	c.1246G>A	p.Ala416Thr	Heterozygote	VUS	PM2	1 (0.7%)
	c.238C>T	p.Arg80Cys	Heterozygote	VUS	PM2	1 (0.7%)
	c.394C>T	p.Arg132Cys	Heterozygote	VUS	PM2	1 (0.7%)
NOTCH3	c.619C>T	p.Arg207Cys	Heterozygote	P	PS4, PM2, PM1, PP3, PP2, PP5	1 (0.7%)
NR2E3	c.119-2A>C		Heterozygote	P	PVS1, PM3, PM2, PS3, PP1, PP5	1 (0.7%)
NRXN1	c.217G>A	p.Glu73Lys	Heterozygote	VUS	PM2, BP1	1 (0.7%)
PAH	c.776C>T	p.Ala259Val	Heterozygote	P	PM3, PP3, PM2, PM5, PM1, PP2, PS3, PP5	1 (0.7%)
	c.631C>A	p.Pro211Thr	Heterozygote	P	PM3, PM2, PM5, PM1, PP4, PP3, PP2, PP5	1 (0.7%)
PCDH19	c.3344G>C	p.Gly1115Ala	Heterozygote	VUS	PM2, PP2	1 (0.7%)
PIGN	c.736A>T	p.Asn246Tyr	Homozygote	VUS	PM2, BP4	1 (0.7%)
	c.364G>C	p.Glu122Gln	Heterozygote	B	BP6, BS1, BS2, BA1	1 (0.7%)
PIGO	c.3163T>C	p.Phe1055Leu	Heterozygote	LB	BS2, BP6, BS1	1 (0.7%)
PLCB1	c.3643C>T	p.Pro1215Ser	Heterozygote	VUS	PM2, PP2, BP4	1 (0.7%)
	c.913G>A	p.Gly305Arg	Heterozygote	VUS	PM2, PP2	1 (0.7%)
POLG	c.3151G>C	p.Gly1051Arg	Heterozygote	P	PS1, PM3, PM2, PM5, PM1, PP3, PP2, PS3, PP1, PP5	2 (1.4%)
	c.1402A>G	p.Asn468Asp	Heterozygote	LP	PM3, PM2, PP2, PS3, PP5	1 (0.7%)
	c.1721G>A	p.Arg574Gln	Heterozygote	P	PS4, PM2, PM5, PM1, PP3, PP2, PP5	1 (0.7%)
	c.823C>T	p.Arg275*	Heterozygote	P	PVS1, PM2, PS4, PP5	1 (0.7%)
	c.1790G>A	p.Arg597Gln	Heterozygote	P	PM3, PM2, PM5, PM1, PP2, PP1, PP5	1 (0.7%)
	c.2446C>G	p.Leu816Val	Heterozygote	VUS	PM2, PP3, PP2	1 (0.7%)
POLG2	c.798T>G	p.Phe266Leu	Heterozygote	VUS	PM2	1 (0.7%)
POLR3A	c.2992C>T	p.Arg998Cys	Heterozygote	VUS	PM2, PP2	1 (0.7%)
	c.91C>G	p.Gln31Glu	Heterozygote	VUS	PM2, PP2	1 (0.7%)
	c.1427_1429del	p.His476del	Heterozygote	VUS	PM2, PM4	1 (0.7%)
POLR3B	c.1648C>T	p.Arg550*	Heterozygote	P	PVS1, PM3, PM2, PS3, PP5	1 (0.7%)
PPT1	c.255_257del	p.Phe85del	Heterozygote	P	PM3, PM2, PM4, PM1, PS3, PP5	1 (0.7%)
PRRT2	c.649dup	p.Arg217Profs*8	Heterozygote	P	PVS1, PM2, PS4, PS3, PP1, PP5	2 (1.4%)
	c.649del	p.Arg217Glufs*12	Heterozygote	P	PVS1, PM2, PS4, PS3, PP5	1 (0.7%)
	c.647C>G	p.Pro216Arg	Heterozygote	VUS	PM2, BP6	1 (0.7%)
	c.883C>T	p.Arg295Trp	Heterozygote	LP	PM2, PM5, PM1, PP3	1 (0.7%)
PTS	c.370G>T	p.Val124Leu	Heterozygote	P	PS1, PM3, PM2, PM1, PP2, PP5	1 (0.7%)
RELN	c.2015C>T	p.Pro672Leu	Homozygote	VUS	PM2, BP6	1 (0.7%)
	c.2015C>T	p.Pro672Leu	Heterozygote	VUS	PM2, BP6	1 (0.7%)
	c.6245G>T	p.Gly2082Val	Heterozygote	VUS	PM2	1 (0.7%)
	c.8798C>T	p.Thr2933Ile	Heterozygote	LB	PM2, BP4, BP6, BS2	1 (0.7%)
RNASEH2B	c.529G>A	p.Ala177Thr	Heterozygote	P	PP1, PM2, PP2, PS3, PP5	1 (0.7%)
	c.898T>C	p.Phe300Leu	Heterozygote	VUS	PP3, PM2, PP2	1 (0.7%)
	c.4G>C	p.Ala2Pro	Heterozygote	VUS	PM2, PP2	1 (0.7%)
SCARB2	c.194A>G	p.Tyr65Cys	Heterozygote	VUS	PP3, BS1	1 (0.7%)
SCN1A	c.1625G>A	p.Arg542Gln	Heterozygote	B	PP2, BP6, BS1, BS2, BA1	1 (0.7%)
	c.5057del	p.Ile1686fs (p.Ile1686Thrfs*29)	Heterozygote	LP	PVS1, PM2	1 (0.7%)
	c.2345C>G	p.Thr782Ser	Heterozygote	LP	PM2, PM5, PM1, PP3, PP2	1 (0.7%)
	c.473A>G	p.Glu158Gly	Heterozygote	LP	PP3, PM2, PM5, PP2	1 (0.7%)
	c.5783G>T	p.Arg1928Leu	Heterozygote	VUS	PM2, PP3, PP2	1 (0.7%)
	c.1193C>T	p.Thr398Met	Heterozygote	LP	PM2, PM1, PP3, PP2, PP5	1 (0.7%)
	c.1837C>T	p.Arg613*	Heterozygote	P	PVS1, PS2, PM2, PP5	1 (0.7%)
SCN2A	c.269C>T	p.Thr90Met	Heterozygote	VUS	PP3, PM2, PP2	1 (0.7%)
SCN3A	c.1393G>A	p.Ala465Thr	Heterozygote	VUS	PM2, PP3, PP2	1 (0.7%)
SCN8A	c.3149G>C	p.Gly1050Ala	Heterozygote	VUS	PM2, PP5, PP2	1 (0.7%)
SCN9A	c.3802G>A	p.Val1268Ile	Heterozygote	VUS	PM2, PP3	1 (0.7%)
	c.1846G>A	p.Gly616Arg	Heterozygote	LP	PP3, PM2, PP5	1 (0.7%)
	c.1744G>A	p.Glu582Lys	Heterozygote	VUS	PM2	2 (1.4%)
	c.1756G>T	p.Gly586Cys	Heterozygote	VUS	PM2, PP3	1 (0.7%)
	c.3391G>T	p.Asp1131Tyr	Heterozygote	VUS	PM2, PP3	1 (0.7%)
	c.58C>T	p.Leu20Phe	Heterozygote	VUS	PP3, PM2	1 (0.7%)
SERAC1	c.1822_1828+10delinsACCAACAGG		Heterozygote	P	PM3, PVS1, PM2, PP5	1 (0.7%)
SLC25A22	c.905C>T	p.Ala302Val	Heterozygote	VUS	PM2	1 (0.7%)
	c.293+3G>A		Heterozygote	VUS	PM2, BP4	1 (0.7%)
	c.731A>G	p.Asn244Ser	Heterozygote	VUS	PM2	1 (0.7%)
SLC46A1	c.340C>T	p.Arg114Cys	Heterozygote	VUS	PP3, PM2	1 (0.7%)
SLC6A19	c.719G>A	p.Arg240Gln	Heterozygote	VUS	PP3, PM2	1 (0.7%)
	c.728C>T	p.Thr243Met	Heterozygote	VUS	PP3, PM2	1 (0.7%)
	c.649G>A	p.Glu217Lys	Heterozygote	VUS	PM2	1 (0.7%)
	c.1606G>A	p.Val536Met	Heterozygote	VUS	PM2, BP6	1 (0.7%)
SMARCA2	c.2898C>G	p.Ile966Met	Homozygote	VUS	PM2, PP2	1 (0.7%)
	c.2381A>G	p.Lys794Arg	Heterozygote	VUS	PM2, PP3, PP2	1 (0.7%)
	c.686_715del	p.Gln229_Gln238del	Heterozygote	VUS	PM2, BP3	1 (0.7%)
	c.3737G>A	p.Arg1246Gln	Heterozygote	VUS	PM2, PP2	1 (0.7%)
SMC1A	c.3649A>G	p.Thr1217Ala	Hemizygote	VUS	PM2, PP3, PP2	1 (0.7%)
SMS	c.536G>A	p.Arg179Gln	Hemizygote	VUS	PM2, PP2, BS2	1 (0.7%)
SPTAN1	c.6310C>T (c.6274C>T)	p.Arg2104Cys (p.Arg2092Cys)	Heterozygote	VUS	PM2, PP2	1 (0.7%)
	c.3779A>G	p.Asn1260Ser	Heterozygote	VUS	PM2, PP2, BP4	1 (0.7%)
	c.7415C>A (c.7316C>A)	p.Thr2472Asn (p.Thr2439Asn)	Heterozygote	VUS	PM2, PP2	1 (0.7%)
SUMF1	c.875A>G	p.Tyr292Cys	Heterozygote	VUS	PP3, PM2	1 (0.7%)
SUOX	c.892C>T	p.Leu298Phe	Heterozygote	LP	PP3, PM2	1 (0.7%)
TBC1D24	c.965+2T>C		Heterozygote	P	PVS1, PM2, PS4, PP5	1 (0.7%)
	c.1453G>A	p.Ala485Thr	Heterozygote	VUS	PM2, BP4	1 (0.7%)
	c.1457G>A	p.Arg486His	Heterozygote	VUS	PM2	1 (0.7%)
	c.641G>A	p.Arg214His	Heterozygote	B	BP6, BS1, BS2	1 (0.7%)
TMEM43	c.520G>A	p.Ala174Thr	Heterozygote	VUS	PM2	1 (0.7%)
TPK1	c.371C>T	p.Thr124Ile	Heterozygote	VUS	PM2, PP2, BS2	1 (0.7%)
TRAK1	c.1549G>A	p.Glu517Lys	Heterozygote	B	BS1, BS2, BP6, BA1	1 (0.7%)
	c.175G>A	p.Asp59Asn	Heterozygote	B	BS2, BP6	1 (0.7%)
TYRP1	c.1133A>G	p.Asn378Ser	Heterozygote	P	PP3, PM3, PM2, BS2, PP5	1 (0.7%)
UNC80	c.9460C>T	p.Arg3154Trp	Heterozygote	VUS	PM2, PP2	1 (0.7%)
	c.1447C>A	p.Leu483Met	Heterozygote	VUS	PM2, PP2	1 (0.7%)
	c.3041+1_3041+2insT (c.3041+2dup)		Heterozygote	VUS	PM2, PP3	1 (0.7%)
VPS13A	c.7096C>T	p.Pro2366Ser	Heterozygote	LB	PM2, BS2, BP4	1 (0.7%)
ZFYVE26	c.4048G>A	p.Glu1350Lys	Heterozygote	VUS	PM2, BS2	1 (0.7%)
	c.6276_6278del	p.Pro2093del	Heterozygote	VUS	PM2, PM4	1 (0.7%)

ACMG: American College of Medical Genetics and Genomics, B: benign, LB: likely benign, P: pathogenic, LP: likely pathogenic, VUS: variant of uncertain significance.

**Table 3:** Genetic variations in pediatric-onset epilepsy patients as determined by WES.

Genes, NM #	Nucleotide Change	Amino Acid Change	Zygosity	ACMG Classification		Number of Patients (%)
ABCD1, NM_000033.4	c.265C>G	p.Arg89Gly	Homozygote	VUS	PM2, PP2	1 (1.5%)
ADGRV1, NM_032119.4	c.13930T>A	p.Phe4644Ile	Heterozygote	VUS	PM2	1 (1.5%)
	c.1127C>G	p.Pro376Arg	Heterozygote	VUS	PM2	1 (1.5%)
ALG13, NM_001099922.3	c.2918G>A	p.Ser973Asn	Hemizygote	VUS	PM2, BP4	1 (1.5%)
ARHGEF10, NM_001308152.2	c.3079+2T>C		Heterozygote	VUS	PM2	1 (1.5%)
ATIC, NM_004044.7	c.291-1G>C		Heterozygote	LP	PVS1, PM2	1 (1.5%)
ATP7B, NM_000053.4	c.3979C>G	p.Leu1327Val	Heterozygote	LP	PM2, PM1, PP3, PP2, PP5	1 (1.5%)
BTD, NM_001323582.1	c.410G>A	p.Arg137His	Heterozygote	P	PM3, PM2, PM5, PM1, PP2, PP5	1 (1.5%)
	c.407A>G	p.Gln136Arg	Heterozygote	VUS	PM2, PM1, PP2	1 (1.5%)
	c.1270G>C	p.Asp424His	Heterozygote	VUS	PP5, PM5, PM1, PP3, PP2, BS2, BA1	1 (1.5%)
CACNA1H, NM_001005407.2	c.5006G>A	p.Arg1675Gln	Heterozygote	VUS	PP3, PM2	1 (1.5%)
	c.5987dupG	p.Thr2003Hisfs*27	Heterozygote	VUS	PM2, PVS1	1 (1.5%)
CAPN3, NM_000070.3	c.2107C>T	p.Leu703Phe	Homozygote	LP	PM2, PM1, PP3, PP2	1 (1.5%)
CDKL5, NM_003159.3	c.1141A>G	p.Thr381Ala	Heterozygote	VUS	PM2, BP4	1 (1.5%)
CHD2, NM_001271.4	c.1250G>A	p.Trp417*	Heterozygote	LP	PVS1, PM2	1 (1.5%)
CLCN1, NM_000083.3	c.2680C>T	p.Arg894*	Heterozygote	LP	PVS1, PM2, PP5	1 (1.5%)
CLN3, NM_000086.2	c.586dup	p.Ala196Glyfs*40	Homozygote	P	PVS1, PM2, PP5	1 (1.5%)
CNTNAP2, NM_014141.6	c.1562T>G	p.Met521Arg	Homozygote	LP	PP3, PM2	1 (1.5%)
CPS1, NM_001122633.3	c.2407C>T	p.Arg803Cys	Heterozygote	LP	PP3, PM2, PM5, PM1, PP5	1 (1.5%)
DLD, NM_000108.5	c.418del	p.His140Thrfs*17	Heterozygote	LP	PVS1, PM2	1 (1.5%)
ERMARD, NM_001278532.2	c.1150delC	p.Gln510Serfs*47	Heterozygote	LP	PVS1, PM2	1 (1.5%)
FGF12, NM_001377292.1	c.487A>G	p.Lys163Glu	Heterozygote	VUS	PM2	1 (1.5%)
FBXO11, NM_025133.4	c.2087-3C>T		Heterozygote	VUS	PM2, BP4	1 (1.5%)
GRIN2D, NM_000836.4	c.3479G>T	p.Gly1160Val	Heterozygote	VUS	PM2, PP2, BP4	1 (1.5%)
GBA, NM_001005741.3	c.1175G>A	p.Arg392Gln	Heterozygote	LP	PM2, PM1, PP3, PM5, PP2	1 (1.5%)
GNB1, NM_002074.5	c.803_805del	p.Asn268del	Heterozygote	VUS	PM2, PM4	1 (1.5%)
KIAA0586, NM_001244189.2	c.74delA (c.38del)	p.Lys25fs*6 (p.Lys13Argfs*6)	Heterozygote	P	PVS1, PM3, PM2, PP5	1 (1.5%)
	c.2129G>A (c.1970G>A)	p.Ser710Asn (p.Ser657Asn)	Heterozygote	VUS	PM2, BP4	1 (1.5%)
KCNA2, NM_004974.4	c.997T>C	p.Phe333Leu	Heterozygote	LP	PM2, PM1, PP3, PP2	1 (1.5%)
KIAA0586, NM_001329947.2	c.2750C>T	p.Thr917Ile	Homozygote	VUS	PM2, BP4	1 (1.5%)
LGI4, NM_139284.3	c.157A>G	p.Thr53Ala	Homozygote	VUS	PM2	1 (1.5%)
LAMA2, NM_001079823.2	c.2451-2A>G		Heterozygote	P	PVS1, PM3, PM2, PP5	1 (1.5%)
MECP2, NM_004992.4	c.379C>G (c.415C>G)	p.Pro127Ala (p.Pro139Ala)	Hemizygote	LP	PM2, PM5, PP3, PM1	1 (1.5%)
MED23, NM_004830.4	c.1984C>T	p.Gln662*	Heterozygote	LP	PVS1, PM2	1 (1.5%)
MTOR, NM_004958.4	c.6676G>T	p.Ala2226Ser	Heterozygote	VUS	PM2, PP2	1 (1.5%)
NHLRC2, NM_198514.4	c.442G>T	p.Asp148Tyr	Heterozygote	LP	PM3, PP3, PM2, PP5	1 (1.5%)
NEXMIF, NM_001008537.3	c.1627_1628del	p.Ile543Hisfs*3	Hemizygote	LP	PVS1, PM2	1 (1.5%)
NAA15, NM_057175.5	c.779A>G	p.Tyr260Cys	Heterozygote	VUS	PM2, PP3, PP2	1 (1.5%)
NDUFAF5, NM_001039375.3	c.174G>A	p.Trp58*	Heterozygote	P	PVS1, PM2, PP5	1 (1.5%)
NPRL3, NM_001243249.2	c.388C>A (c.463C>A)	p.His130Asn (p.His155Asn)	Heterozygote	VUS	PM2, PP3, PP2	1 (1.5%)
PIGS, NM_033198.4	c.1631C>G	p.Thr544Ser	Homozygote	VUS	PM2, BP4	1 (1.5%)
PCCA, NM_000282.4	c.230G>A	p.Arg77Gln	Heterozygote	P	PM3, PP3, PM2, PM5, PM1, PP2, PP5	1 (1.5%)
PTCHD1, NM_173495.3	c.681G>C	p.Glu227Asp	Hemizygote	VUS	PM2	1 (1.5%)
PIK3CA, NM_006218.4	c.3010A>G	p.Met1004Val	Heterozygote	LP	PM2, PM5, PP3, PP2	1 (1.5%)
RELN, NM_173054.3	c.3296G>A	p.G1099D (p.Gly1099Asp)	Heterozygote	VUS	PM2	1 (1.5%)
SCN1A, NM_001165963.4	c.4220G>A	p.Arg1407Gln	Heterozygote	LP	PM2, PM1, PP3, PP2	1 (1.5%)
	c.4003-2_4008del		Heterozygote	LP	PVS1, PM2	1 (1.5%)
SCN8A, NM_001177984.3	c.4762T>C (c.4885C>T)	p.Phe1588Leu (p.Arg1629Cys)	Heterozygote	LP	PP3, PM2, PM1, PP2	1 (1.5%)
	c.2188T>C	p.Phe730Leu	Heterozygote	VUS	PM2, PP3, PP2	1 (1.5%)
	c.508A>T	p.Thr170Ser	Heterozygote	LP	PP3, PM2, PP2	1 (1.5%)
SCN9A, NM_001365536.1	c.1699G>A	p.Glu567Lys	Homozygote	VUS	PM2, PP3	1 (1.5%)
SOS1, NM_005633.4	c.2155G>A	p.Gly719Arg	Heterozygote	VUS	PM2, BP4	1 (1.5%)
SET, NM_003011.4	c.1A>T	p.Met1?	Heterozygote	VUS	PM2, PVS1	1 (1.5%)
SKI, NM_003036.4	c.1780del	p.Leu594Tyrfs*34	Heterozygote	LP	PVS1, PM2	1 (1.5%)
SETBP1, NM_015559.3	c.2426A>T	p.Gln809Leu	Heterozygote	VUS	PM2	1 (1.5%)
SEPSECS, NM_016955.4	c.289C>T	p.Arg97*	Heterozygote	P	PVS1, PM2, PP5	1 (1.5%)
SRPX2, NM_014467.3	c.202G>A	p.Glu68Lys	Hemizygote	VUS	PM2, BS2	1 (1.5%)
TRIM8, NM_030912.3	c.1265C>A	p.Thr422Lys	Heterozygote	VUS	PM2	1 (1.5%)
THOC2, NM_001081550.2	c.1109C>T	p.Pro370Leu	Homozygote	VUS	PM2, PP2, BP4	1 (1.5%)
TSC2, NM_000548.5	c.169C>T	p.Arg57Cys	Heterozygote	VUS	PP3, PM2	1 (1.5%)
WWOX, NM_001291997.2	c.645C>G (c.984C>G)	p.Tyr215* (p.Tyr328*)	Homozygote	LP	PVS1, PM2	1 (1.5%)
WDR62, NM_173636.5	c.1576G>A	p.Glu526Lys	Homozygote	LP	PM3, PM2, BP6, PP5	2 (3.0%)

ACMG: American College of Medical Genetics and Genomics, P: pathogenic, LP: likely pathogenic, VUS: variant of uncertain significance. # indicates number.

## Data Availability

The authors confirm that the data supporting the findings of this study are available within the article and its Supplementary Materials.

## Supplementary Materials

The following supporting information can be downloaded at https://www.mdpi.com/article/10.3390/genes16060624/s1, Table S1: Epilepsy panel genes.
