# Supplementary material for: Clinical and Genetic Spectrum of Patients with Pediatric-Onset Epilepsy: Insights from a Single-Center Study
Source: Genes (Basel). 2025 May 24;16(6):624. doi: 10.3390/genes16060624 (PMC12192223; doi:10.3390/genes16060624)
Supplement: Supplementary file 1 [file genes-16-00624-s001.zip › genes-3609104-supplementary.pdf]

Supplementary Table S1. Epilepsy panel genes

ABAT	CHD2	ETFB	IQSEC2	PCDH19	SLC46A1
ABCA2	CHRNA2	ETFDH	KCNA1	PGK1	SLC6A1
ABCD1	CHRNA4	ETHE1	KCNMA1	PHF6	SLC6A8
ADAM22	CHRNB2	FA2H	KCNQ2	PIGA	SLC9A6
ADAR	CLCN2	FAM126A	KCNQ3	PIGN	SMARCA2
ADSL	CLCN4	FARS2	KCNQ5	PIGO	SMC1A
AFG3L2	CLN3	FDFT1	KCNT1	PIGV	SMS
AGA	CLN5	FH	KCTD7	PLCB1	SNAP25
AIFM1	CLN6	FLNA	KDM5C	PLP1	SOX10
AIMP1	CLN8	FOLR1	KIAA2022	PNKP	SPTAN1
ALDH3A2	CNKSR2	FOXG1	KIF1A	PNPO	ST3GAL3
ALDH5A1	CNPY3	FOXRED1	L2HGDH	POLG	ST3GAL5
ALDH7A1	CNTNAP2	FUT8	LGI1	POLR3A	STXBP1
ALG13	COL4A1	GABRA1	LMNB1	POLR3B	SUMF1
AMACR	COX15	GABRB3	LRPPRC	PPP3CA	SUOX
AMT	COX6B1	GABRG2	MAGI2	PPT1	SYN1
ANKRD11	CPT2	GALC	MARS2	PRICKLE1	TAF1
AP3B2	CSF1R	GAMT	MBD5	PRODH	TBC1D24
AP4B1	CSTB	GCDH	MBOAT7	PRRT2	TBCD
AP4E1	CTC1	GCH1	MECP2	PSAP	TBCE
AP4M1	CTSD	GFAP	MED12	PTS	TBCK
AP4S1	CUL4B	GFM1	MED17	QDPR	TBL1XR1
ARG1	CYP27A1	GJC2	MEF2C	RAB39B	TCF4
ARHGEF9	D2HGDH	GLB1	MFSD8	RELN	TPK1
ARID1B	DARS2	GLDC	MLC1	RMND1	TPP1
ARSA	DCX	GLRB	MOCS1	RNASEH2A	TRAK1
ARX	DDC	GNE	MTFMT	RNASEH2B	TREX1
ASAH1	DDX3X	GOSR2	MTHFR	RNASEH2C	TSC1
ASNS	DHFR	GPHN	NBEA	RNASET2	TSC2
ASPA	DNAJC5	GRIA3	NDST1	ROGDI	TTC19
ATP13A2	DNM1	GRIA4	NDUFAF3	SAMHD1	TUBB4A
ATP1A3	DNM1L	GRIK2	NDUFAF5	SCARB2	UBA5
ATRX	DOCK7	GRIN1	NDUFAF6	SCN1A	UBE2A
BRAT1	DPYD	GRIN2A	NDUFS2	SCN1B	UBE3A
BTD	DPYS	GRIN2B	NDUFS4	SCN2A	UNC80
C12ORF57	DYRK1A	GRN	NDUFS6	SCN3A	VPS13A
CACNA1A	EARS2	HACE1	NDUFS7	SCN8A	WDR45
CACNA1D	ECM1	HCN1	NDUFS8	SCN9A	WWOX
CACNA1E	EFHC1	HCN2	NDUFV1	SCO1	YY1
CACNA1H	EIF2B1	HEPACAM	NFU1	SDHAF1	ZEB2
CACNB4	EIF2B2	HIBCH	NHLRC1	SERAC1	ZFYVE26
CASK	EIF2B3	HNRNPU	NOTCH3	SERPINI1	
CASR	EIF2B4	HSD17B10	NRXN1	SLC19A3	
CC2D1A	EIF2B5	HTRA1	NUBPL	SLC25A15	
CDKL5	EPM2A	HTT	OFD1	SLC25A22	
CERS1	ETFA	ICK	OPHN1	SLC2A1	
